# Encephalocele as a complication post-craniosynostosis surgery

**DOI:** 10.1093/jscr/rjae442

**Published:** 2024-07-11

**Authors:** Imad Talahma, Yousef S Abuzneid, Ameer Ismail, Lina Alqam, Amaal Mufreh, Bara Abu Shama'a

**Affiliations:** Neurosurgery Department, Neuro-Spine Center, Palestine Red Crescent Society Hospital, Hebron, State of Palestine; Neurosurgery Department, Palestine Polytechnic University Faculty of Medicine, Hebron, State of Palestine; Neurosurgery Department, Neuro-Spine Center, Palestine Red Crescent Society Hospital, Hebron, State of Palestine; Palestine Polytechnic University Faculty of Medicine, Hebron, State of Palestine; Palestine Polytechnic University Faculty of Medicine, Hebron, State of Palestine; Palestine Polytechnic University Faculty of Medicine, Hebron, State of Palestine; Palestine Polytechnic University Faculty of Medicine, Hebron, State of Palestine

**Keywords:** encephalocele, craniosynostosis, complication, surgery, case report

## Abstract

Duraplasty is one of the most common neurosurgical procedures which complications include iatrogenic pseudomeningocele, which is common, but ossification of pseudomeningocele following cranial surgery is a rare event. We present a case of a 2-year-old male patient who came to our hospital with a huge bulge in his head and weakness in the right arm and leg. He had a history of sagittal craniosynostosis with a postoperative cranioplasty complication of left parital pseudomeningocele. He underwent a duraplasty, but the bulge recurred with failed cerebrospinal fluid aspiration and external ventricular drain, changing in size periodically. Computed tomography showed that the bulge was a median and left paramedian parital encephalocele, so encephalocele with ossification was diagnosed and a cranioplasty was done. This case highlights that iatrogenic encephaloceles with ossification can develop after duraplasty repair in the parital region. Also, if a postoperative pseudomeningocele changes in size or consistency, clinicians should look for ossification.

## Introduction

Premature union of one or more cranial sutures is known as craniosynostosis [1]. Pseudomeningocele is an extradural collection of cerebrospinal fluid (CSF) that communicates with CSF spaces around the brain or spinal cord [2]. Iatrogenic pseudomeningocele is a common complication seen frequently postoperatively, but ossification of pseudomeningocele is an uncommon situation; only four cases have been documented in the literature to date. Moreover, encephalocele post-craniosynostosis surgery is a very rare entity [3]. Here, we describe a case of a pediatric patient who initially presented with craniosynostosis at the sagittal suture and underwent cranioplasty, then duraplasty for pseoudamoningocele, followed by the development of an encephalocele with ossification.

## Case presentation

A 2-year-old male child was admitted as a case of post-operative cranioplasty complication with brain bulging. The patient was born at full term with normal weight, length, and head circumference.

**Figure 1:**
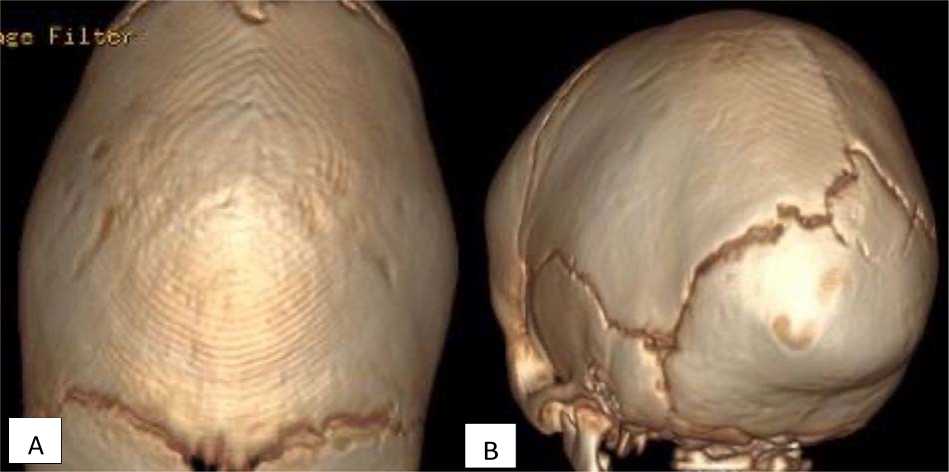
Initial computed tomography scan (CT) scan obtained when the patient was 3 months old showing sagittal craniosynostosis. (A) Superior axial view and (B) posterior view.

At the age of one month, the patient was diagnosed with sagittal craniosynostosis (Fig. 1), and by the age of 4 months, he underwent a strip craniectomy in another hospital to treat it without any acute complications; however, 2 weeks later his family noticed a bulging.

**Figure 2:**
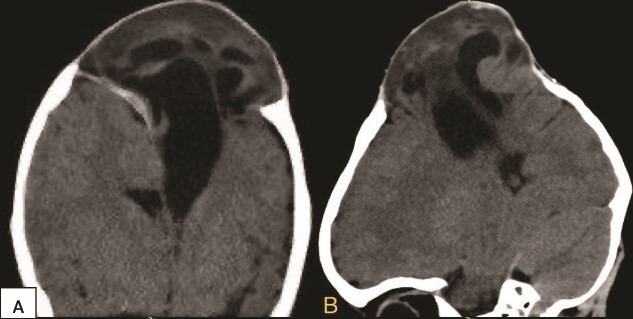
Preoperative coronal (A) and sagittal (B) brain CT scan demonstrating the encephalocele.

Upon seeking medical advice, the patient was found to have a left parietal pseudomeningocele and later he underwent a duraplasty with evacuation of the pseudomeningocele but months later, a bulge reappeared. Multiple follow ups were done through which CSF aspiration by needle was performed with the hope of CSF to stop spontaneously. At the age of 2 years, an external ventricular drain (EVD) was inserted without significant improvement and a complain of right arm and leg weakness was noticed, so he was referred to our hospital for further treatment. Hence, a CT scan was done (Fig. 2), revealing a median and left paramedian parietal encephalocele causing detorsion and compression of the sagittal sinus, and part of the primary motor area was extending into the encephalocele region.

**Figure 3:**
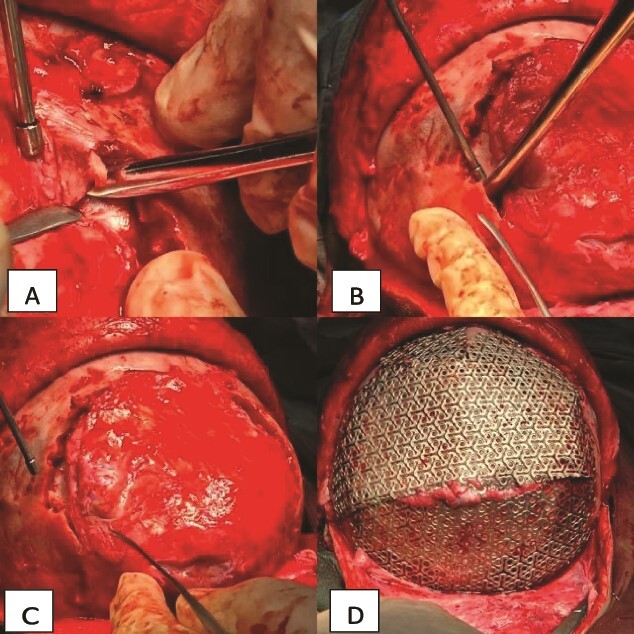
Intraoperative view of the case. (A) Showing the ossification and adhesion of the bone to the brain tissue. (B) Showing the cutting technique used to separate the bone. (C) Showing the brain tissue separated from the bone and free. (D) Showing the cranial mesh inserted to cover the cranial defect and protect the brain tissue.

**Figure 4:**
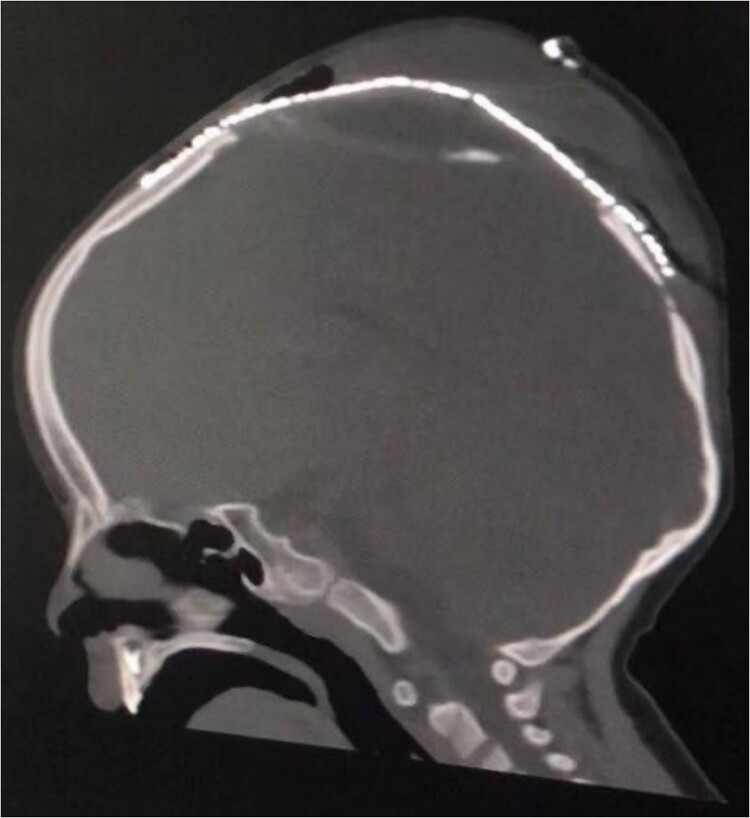
Postoperative sagittal brain CT demonstrating that the brain was inside the cranium and covered by the cranial mesh, preventing further bulging.

Furthermore, a decision to perform a cranioplasty was made immediately. In the operation the previous bicoronal flap was reopened, isolating the encephalocele. It was found a newly formed tough ring of bone encircling the base of the encephalocele, with the edges of this bone intruding into the brain tissue with significant adhesions, indicating abnormal bone growth around the affected area. Carefully, the newly formed bone was removed to reach the healthy bone of the skull. A temporary EVD was used alongside mannitol administration and hyperventilation to reduce the CSF. The sac was pushed inside and subsequently, a decision for 3D cranial mesh (Fig. 3) closure was made to cover the defect in the cranium for finally close the incision normally. Postoperative CT scan was done (Fig. 4), demonstrating that the brain went inside with no postoperative complications. There was notable improvement in the motor functions of the right limbs and absence of spasticity in the right upper limb. Two days later the patient was discharged home, active and able to walk without assistance.

## Discussion

Duraplasty aims to ensure dural closure in cranial procedures, but its complications include CSF leakage, pseudomeningocele, and rarely encephaloceles [4]. Encephalocele is a herniation of the brain with meninges through the cranial cavity. They are mostly congenital but can be iatrogenic after vacuum extraction or as a late complication after cranial reconstruction surgery [5]. Treatment of encephalocele is surgical, aiming to remove bulging necrotic tissue and ease living tissue into the skull, with drainage of excessive fluid and cutting the skull to relieve pressure [6].

Pseudomeningocele ossification is frequently reported after lumbar surgeries but rarely in cranium. Based on our knowledge, this is the fifth case reported in the literature, and the first after craniosynostosis repair. Most patients are asymptomatic but neurological defects may occur. The reports lack clear mechanisms of ossification. One of the hypotheses is metaplasia of soft tissue surrounding the CSF collection to form cartilage and bone. Another explanation hypothesized that residual postoperative hemorrhage of tissue initiates an inflammatory reaction that catalyzes it [3]. Usually, postoperative pseudomeningocele is treated conservatively, resolving spontaneously within 7–14 days before surgery with duraplasty is considered. In cases of an enlarging pseudomeningocele, CSF diversion using endoscopic third ventriculostomy, or shunting is performed [3]. Our patient underwent duraplasty in another hospital for occipitoparietal meningoencephalocele but developed a pseudomeningocele after surgery. Multiple CSF aspirations failed, so an EVD was inserted for drainage with no improvement. There were periods of spontaneous reduction in the size of the pseudomeningocele, but recurrent increase in size always ensued.

According to a case by Danial Lewis *et al.* in 2022 [3], clinicians should investigate ossification if a postoperative pseudomeningocele changes in size or consistency.

CT scan is indicated to confirm the diagnosis of ossification. When ventriculoperitoneal shunt was planned for our patient, a CT scan was performed, showing an exceptionally rare complication of brain bulging outside the cranium with an ossified ring at the base. Iatrogenic encephalocele with ossification was diagnosed and emergent cranioplasty with brain tissue insertion, after reducing the edema, was performed, followed by cranial mesh placement to cover the cranial defect, and protect the brain tissue.

Based on our literature review, intradiploic meningoencephalocele after craniosynostosis repair was the only reported case presented as a child mimicking our case [7]. However, ours is the first iatrogenic encephalocele after duraplasty repair in the occipitoparietal region and the first with ossification causing pressure on brain tissue, presenting as weakness of right upper and lower limbs. Furthermore, after urgent cranioplasty, we put back the brain tissue inside and closed it properly with cranial mesh to prevent recurrence.

## Conclusion

This case demonstrated that iatrogenic encephalocele can happen after procedures of craniosynostosis repair, which may include cranial vault remodeling and craniotomy. These can be complicated by dural fistula, but spontaneous closure may occur; if not, encephalocele and tissue necrosis can develop. Then, emergent cranioplasty is recommended to bring the brain inside skull with tight coverage by artificial bone or metallic mesh to prevent recurrence. Once the neurological deficits begin, urgent reduction must be considered. Hence, follow up and serial CT scans are important.
